# Impact of ignoring sampling design in the prediction of binary health outcomes through logistic regression: evidence from Malawi demographic and health survey under-five mortality data; 2000-2016

**DOI:** 10.1186/s12889-023-16544-4

**Published:** 2023-08-31

**Authors:** Tsirizani M. Kaombe, Gracious A. Hamuza

**Affiliations:** 1 Department of Mathematical Sciences, School of Natural and Applied Sciences, University of Malawi, Zomba, Malawi; 2 National Statistical Office of Malawi, Zomba, Malawi

**Keywords:** Under-five mortality rate, DHS data, Survey design effect, Logistic regression, Bias

## Abstract

The birth and death rates of a population are among the crucial vital statistics for socio-economic policy planning in any country. Since the under-five mortality rate is one of the indicators for monitoring the health of a population, it requires regular and accurate estimation. The national demographic and health survey data, that are readily available to the puplic, have become a means for answering most health-related questions among African populations, using relevant statistical methods. However, many of such applications tend to ignore *survey design effect* in the estimations, despite the availability of statistical tools that support the analyses. Little is known about the amount of inaccurate information that is generated when predicting under-five mortality rates. This study estimates and compares the bias encountered when applying unweighted and weighted logistic regression methods to predict under-five mortality rate in Malawi using nationwide survey data. The Malawi demographic and health survey data of 2004, 2010, and 2015-16 were used to determine the bias. The analyses were carried out in R software version 3.6.3 and Stata version 12.0. A logistic regression model that included various bio- and socio-demographic factors concerning the child, mother and households was used to estimate the under-five mortality rate. The results showed that accuracy of predicting the national under-five mortality rate hinges on cluster-weighting of the overall predicted probability of child-deaths, regardless of whether the model was weighted or not. Weighting the model caused small positive and negative changes in various fixed-effect estimates, which diffused the result of weighting in the fitted probabilities of deaths. In turn, there was no difference between the overall predicted mortality rate obtained using the weighted model and that obtained in the unweighted model. We recommend considering survey cluster-weights during the computation of overall predicted probability of events for a binary health outcome. This can be done without worrying about the weights during model fitting, whose aim is prediction of the population parameter.

## Introduction

The mortality rate of children aged zero to fifty nine months is a useful indicator for monitoring national and global health targets [1, 16, 32]. Therefore, estimation of the total number of deaths observed in the under-five age group requires robust and reliable techniques, to obtain accurate approximation for policy decisions [13, 16]. It has been reported that there is weak registration of vital systems and high rates of under-reporting deaths at health facilities in sub-Saharan African nations [23, 28, 55]. Most estimations of under-five mortality rate in the region are based on information gathered from national surveys, such as the demographic and health survey (DHS) data [21, 22, 42, 46]. This is what necessitates the use of survey design-based statistical analyses, such as sampling weights, for accurate estimates [5, 19, 20, 40, 44, 53]. A sample weight is the inverse of the probability of a subject being included in the sample. This indicates the number of subjects in the population that each sampled unit represents. During the regression analysis, the subject’s weight is introduced as a functional of covariates in the model that is applied on survey data, to compensate for the use of unequal sample inclusions, non-response, and under-coverage of sampling frame [7, 11, 36, 42, 50, 57].

However, the *survey design effect* is ignored in most applications of regression methods used to estimate the under-five mortality rate in sub-Saharan Africa, which potentially biases the estimates and predictions [18, 41, 43, 52]. This problem was also found to be true for other studies that analysed binary health outcomes apart from mortality. For example, the presence or absence of diabetes [48], diarrhoea [33], schistosomiasis [14, 31], and malaria [29], among other diseases in patients. This trend could reflect the unavailability of studies that demonstrate the technical use of survey designs, when applying regression methods to binary health data. It might also be due to the fact that most of the reviewed studies aimed to identify risk factors of the concerned health outcomes, rather than predicting the extent of the physical condition itself, which could be achieved in the population any way [9, 17, 54]. There is a dearth of literature on the bias one would commit should the national under-five mortality rate or other binary response health data be predicted from a large nationwide survey without regard of the *design effect*. This present study therefore estimates the bias a researcher might commit when predicting the under-five mortality rate using survey weighted and unweighted logistic regression methods. A bias in the estimation of under-five mortality rate is the discrepancy between the rate estimated through random samples and the actual rate reported in routine observations. One would expect the difference between the two values to be zero, in which case the estimator applied on the survey data is said to be unbiased [39]. The present study uses three recent demographic and health survey (DHS) datasets in Malawi for the years 2004, 2010, and 2015-16 and official under-five mortality rates reported by the respective DHS to compute the bias. Various sample sizes of each DHS dataset are used, in order to account for sample size effect in the bias estimation.

It is important for health researchers to know about the worthiness of survey design information in the binary regression estimation methods, so they can make informed decisions. They need to determine the merits of and demerits of including survey weights when analysing binary health data using regression techniques [11]. Knowledge of the mortality estimate bias from this study will give evidence for considering survey design in health research that involves prediction of some binary ouctomes. The rest of the article is organised as follows: in Section “Methods” statistical methods and data used in this study are presented; Section “Results” shows results; while in Section “Discussion” the findings are discussed. Finally, Section “Conclusion” is the conclusion of the paper.

## Methods

### Data

This study used secondary data for children obtained from the *kids’ records* file of the Malawi Demographic and Health Survey (MDHS). These data sets were gotten from the surveys conducted between October 2004 and January 2005, then June to November 2010, and lastly, between October 2015 and February 2016. The MDHS uses two-stage stratified cluster random sampling, where 522, 849, and 850 clusters (that is, enumeration areas) were sampled from across the country respectively, at the initial stage. At the second stage, 15,091, 27,345, and 27,516 households were sampled respectively from the initial selected clusters, using the rural and urban stratifications [30, 34, 35]. An enumeration area (cluster or village) is a geographic area that covers, for example, between 0 and 954 households or an average of 235 households [24]. Each cluster contains information about its location, strata (urban or rural), an estimated number of residential households, as well as a sketch map showing the boundaries, location of buildings, and other landmarks. The clusters determined during the 1998 population and housing census constituted a sampling frame for primary sampling units in the 2004 MDHS, and those defined in the 2008 national census formed the frame for the 2010 and 2015-16 MDHSs [30, 34, 35]. The children who were aged below five years in the sampled households formed the sample for this study. A total of 10,914, 19,967, and 17,286 under-five children were sampled for study in the 2004, 2010, and 2015-16 MDHSs, respectively. The mothers or adult caregivers aged between 15 to 49 years provided birth histories of the children, including the mortality data.

The response variable for this study was whether or not a child under five years of age from any household had died in the last five years preceding the survey. To compute the probability of a child dying before the age of five years, various risk factors that concern the child, mother and household environment were used based on literature. These are: birth weight, birth order, sex, whether the birth was singleton or multiple, type of delivery (regular or caesarean), maternal age, education, and occupation, place of residence, including region of the country, place of delivery, preceding birth interval, contraceptive use, and antenatal clinic (ANC) visits during pregnancy [3, 8, 12, 17, 18, 26, 49]. However, the child birth weight and ANC visits variables had at least one-third missing values, hence they were not included in the fitted models. The data management and model fitting were done in STATA software version 12.0, while the calculations of predicted national under-five mortality rate and standard errors were carried out using R software version 3.6.3. In addition, the raw data of under-five mortality rates of 133, 112, and 63 deaths per 1000 live births reported in the respective MDHS reports [30, 34, 35] were used for comparisons with the model-based estimates in this study. The MDHS data that were used in this study can be accessed by users for free at  www.DHSprogram.com..

### Unweighted logistic regression model and predicted mortality rate

Consider a binary outcome variable *Y*, that indicates whether or not a child died on or before attaining the age of 5 years in a household during the last five years of the survey. Let $y_{ic}$ be the observed death outcome for the *i*-th child in *c*-th cluster, $i=1,2,...,n; c = 1,2,...,K$, where $y_{ic}=1$ if the child died, and $y_{ic}=0$ if the child was alive. Further, let $\pi _{ic}=P(Y=1)$ be the probability that the *i*-th child dies in the household from the *c*-th cluster. Then, the total number of deaths in *c*-th cluster given the probability of death and sample size, i.e. $\sum (Y=1|n,\pi _{ic})$ is a $Binomial(n,\pi _{ic})$ random variable, with mass function $f(y_{ic};n,\pi _{ic}) = \exp \left[ y_{ic}\log (\frac{\pi _{ic}}{1-\pi _{ic}})+ n\log (1-\pi _{ic})+\log {{n}\atopwithdelims (){y_{ic}}}\right]$. In addition, let $x^{T}_{ick}=(1,x_{ic1},x_{ic2},...,x_{icp})$ be a vector of explanatory variables observed on the *i*-th child, who is in cluster *c*, where $x_{ic0}=1$ and $k = 1,2,...,p$ is the number of regression coefficients. Therefore, the unweighted conditional probability of a child dying given the covariates $\textbf{x}$, i.e. $\pi _{ic}(\textbf{x})=P(Y=1|\textbf{x})$, relates with the covariates through a logistic function, given by:

1 $$\begin{aligned} \pi _{ic}(\textbf{x})=P(Y=1|\textbf{x})=\frac{exp(\beta ^T\textbf{x})}{1+exp(\beta ^T\textbf{x})}, \end{aligned}$$

where $\beta ^{T}=(\beta _0,\beta _1,...,\beta _p)$ is a vector of regression coefficients and $\textbf{x}=(1,x_{ic1},x_{ic2},...,x_{icp})^{T}$ is a vector of covariates observed on the *i*-th child from *c*-th cluster.

From the relation in Eq. (1), the unweighted logistic regression model with logit link is derived as follows:

2 $$\begin{aligned} \log \left( \frac{\pi _{ic}(\textbf{x})}{1-\pi _{ic}(\textbf{x})}\right) =\beta ^T\textbf{x}=\beta _0 + \beta _1 x_{ic1} + ...+\beta _p x_{icp}. \end{aligned}$$

The maximum likelihood (ML) estimates, $\hat{\beta }$ for the model in Eq. (2) are obtained by multiplying values of probabilities in the mass function $f(y_{ic})$ for all children, and then taking the logarithm of the result. Thereafter, the partial derivatives of the log-likelihood function with respect to $\beta$ are derived, and then equated to zero, from which the ML estimates, $\hat{\beta }$ are solved. Numerical techniques are used to process the solutions, because the equations for the derivatives of the log-likelihood function are not in closed forms [6]. The ML estimate $\hat{\beta }$ for model (2) is interpreted as the change in logarithm of adjusted odds of death of a child as a result of the change in the level of the covariate *X*, while controlling for the other variables in the model. Alternatively, $\hat{\beta }$ can be exponentiated to get $exp(\hat{\beta })$, which is interpreted as the ratio of adjusted odds of deaths of a child when comparing one level of *X* to the other.

Now, the overall unweighted predicted probability of death for all the under-five children in the country was estimated by taking the average of all the fitted probabilities for the model in Eq. (2), as follows:

3 $$\begin{aligned} \hat{\pi }(\textbf{x})=\frac{\sum _{i=1}^{n_{c}}\sum _{c=1}^{K}\hat{\pi }_{ic}}{\sum _{c=1}^{K}n_{c}}, \end{aligned}$$

which is the usual point estimate of the rate of under-five deaths across all clusters, where $\hat{\pi }_{ic}(\textbf{x})=\frac{exp(\hat{\beta }^T\textbf{x})}{1+exp(\hat{\beta }^T\textbf{x})}$ is the fitted probability of death of the *i*-th child in *c*-th cluster given the covariates information $\textbf{x}$ for that child, and given the fitted model. From the probability theory of sampling distribution of sample proportions, the unweighted variance of $\hat{\pi }(\textbf{x})$ was estimated by:

4 $$\begin{aligned} var\left( \hat{\pi }(\textbf{x})\right) =\frac{\hat{\pi }(\textbf{x})(1-\hat{\pi }(\textbf{x}))}{\sum _{c=1}^{K}n_{c}}. \end{aligned}$$

The unweighted measures in Eqs. (3) and (4) can be implemented following either the unweighted model in Eq. (2) or the weighted model that is presented in the next section, because computation of predicted mortality will have to be done separately once the fitted probabilities from the logit model are obtained. The square root of the variance in Eq. (4) provided the standard error of the predicted under-five mortality rate in Eq. (3).

Upon obtaining the national estimate of under-five mortality rate in Eq. (3), the bias of the estimate was computed by:

5 $$\begin{aligned} bias(\hat{\pi }(\textbf{x}))=\hat{\pi }(\textbf{x})-\pi , \end{aligned}$$

where $\hat{\pi }(\textbf{x})$ is the predicted mortality rate obtained in Eq. (3) and $\pi$ is the raw under-five mortality rate given in the particular MDHS report. As alluded to earlier, the models and all other computations stated above were re-done using the three recent MDHS data of 2004, 2010, and 2015-16 in order to confirm the findings. In addition, the usefulness of survey weights in a regression method may depend on a survey sample size [15]. Hence, the above processes were repeated using 75%, 50%, and 25% of the MDHS sample size. This was selected through the same cluster sampling method with the aid of the Stata software function bsample applied on the cluster identification variable, in order to account for the effect of the sample size in the bias estimation [51]. The compuations for statistics in Eqs. (3-5) were implemented in using R software version 3.6.3, while model in Eq. (2) was fitted to the data using Stata software version 12.0.

### Survey weighted logistic regression model and predicted mortality rate

Using the structure of the data presented in Section “Unweighted logistic regression model and predicted mortality rate”, let $w_{ic}=\frac{N_c}{n_c}$ be the sampling weight for the *i*-th child in cluster *c*, who was in a rural or urban stratum, with $N_c$ denoting the population of under-five children in cluster *c* as per proxy census, and $n_c$ the selected number of children in cluster *c*. Based on the 2018 population and housing census, the country had 2, 552, 406 children aged below five years, who were located in 18, 772 enumeration areas (or clusters), that were equally sized [25]. This means that the population of children who were aged five years or below was estimated to be around $N_c = \frac{2,555,406}{18,772}=135.969$ per cluster at the time of the 2015-16 MDHS. Now, considering the under-five children sampled $n_{c}$ in the cluster *c*, this implies that each selected child in the 2015-16 MDHS represented information for $w_{ic}=\frac{135.969}{n_{c}}$ children in their area, depending on the cluster sample size. On the other hand, there were 2, 370, 011 under-five children in Malawi around 2008, who were distributed in 12, 631 equally sized enumeration areas (or clusters) [24]. Hence, the population of under-five children at the time of the 2004 or 2010 MDHS was around $N_c = \frac{2,370,011}{12,631}=187.634$ per cluster. Therefore, each sampled child in the 2004 or 2010 MDHS represented $w_{ic}=\frac{187.634}{n_{c}}$ children in their location. Then, the weighted conditional probability of death of *i*-th child given the covariates **x** is:

6 $$\begin{aligned} \pi _{ic}(\textbf{x})=P(Y=1|\textbf{x})=\frac{exp(\beta ^T\textbf{x}w_{ic})}{1+exp(\beta ^T\textbf{x}w_{ic})}, \end{aligned}$$

with the rest of the quantities defined as in Eq. (1). Therefore, the counterpart weighted logit model [56] to model in Eq. (2) is given by:

7 $$\begin{aligned} \log \left( \frac{\pi _{ic}(\textbf{x})}{1-\pi _{ic}(\textbf{x})}\right) =\beta ^T\textbf{x}w_{ic}=\left[ \beta _0 + \beta _1 x_{ic1} + ...+\beta _p x_{icp}\right] w_{ic}, \end{aligned}$$

where $w_{00}=1$ and the rest of the computations depended on the cluster to which the child belonged. The likelihood function construction process is similar to the one given in Section “Unweighted logistic regression model and predicted mortality rate” for unweighted model. As stated before, this survey weighting ensured that each child’s contribution to the model’s likelihood function took into account the sampling weight for that child, so as to balance off the unequal sample selection, non-response, or under-coverage of the sampling frame between clusters in the computation of the ML estimates [7, 11, 36, 50].

The Stata package survey function svy was used to implement the weighting scheme during model fitting. The jackknife technique was applied to compute standard errors of the regression coefficients’ estimates for the weighted logit model in Eq. (7) [27]. In an ideal case, a hypergeometric probability distribution for the observed number of under-five deaths per cluster was supposed to be assumed instead of the binomial probability distribution, since the sampling of the children was done without replacement and the population became finite as sampling continued. Therefore, the standard error of the binomial response variable, i.e. $\sqrt{n\pi _{ic}(\textbf{x})(1-\pi _{ic}(\textbf{x}))}$ was supposed to be multiplied with the finite population correction (FPC) factor, $FPC=\sqrt{\frac{N-n}{N-1}}$ to make it equivalent to the standard error of the hypergeometric random variable. However, we ignored the *FPC* factor in the computations in this study, as its value was approximately 1 for each survey, i.e. $FPC_{(2015)}=\sqrt{\frac{2,552,406-17,286}{2,552,406-1}}=0.9966$ based on 2015-16 MDHS and 2018 national census, while $FPC_{(2004)}=\sqrt{\frac{2,370,011-10,914}{2,370,011-1}}=0.9976$ based on 2004 MDHS, and $FPC_{(2010)}=\sqrt{\frac{2,370,011-19,967}{2,370,011-1}}=0.9958$ on 2010 MDHS and 2008 census.

Now, upon obtaining the ML estimates and fitted probabilities of deaths from the unweighted logit model in Eq. (2) or the weighted logit model in Eq. (7), one may wish to consider the weights at cluster level during the computation of overall predicted mortality rate. This requires getting cluster-specific unweighted death rates $\hat{\pi }_{c}(\textbf{x})$ first through the method of Eq. (3). Thereafter, the national weighted under-five mortality rate can be estimated by considering each cluster’s weight as follows:

8 $$\begin{aligned} \hat{\pi }^{*}(\textbf{x})=\frac{\sum _{c=1}^{K}\hat{\pi }_{c}(\textbf{x})(\frac{n_{c}}{\bar{m}})}{K}, \end{aligned}$$

where $\bar{m}=\frac{\sum _{c=1}^{K}n_{c}}{K}$ is the average number of sampled children per cluster, $\hat{\pi }_{c}=\frac{\sum _{i=1}^{n_{c}}\hat{\pi }_{i}}{n_{c}}$ is the cluster-specific estimated under-five death rate, in which $\hat{\pi }_{i}$ are fitted probabilities of death obtained from the fitted model, and $\frac{n_{c}}{\bar{m}}$ is the weighting term per cluster. The weighted variance of the overall mortality rate can be computed using the usual basic formula for variance of a random quantity, but now with the weighting term squared, as follows:

9 $$\begin{aligned} var(\hat{\pi }^{*}(\textbf{x}))=\frac{1}{K}\sum \limits _{c=1}^{K}\frac{\left( \hat{\pi }_{c}(\textbf{x})-\hat{\pi }^{*}(\textbf{x})\right) ^{2}}{K-1}\left( \frac{n_{c}}{\bar{m}}\right) ^{2}, \end{aligned}$$

where $\hat{\pi }_{c}(\textbf{x})$ is the estimated under-five death rate in cluster *c*, and $\hat{\pi }^{*}(\textbf{x})$ the weighted estimated national under-five mortaility rate in Eq. (8). After obtaining the weighted overall mortality estimate, the computation of bias in the mortality estimate was done as in Section “Unweighted logistic regression model and predicted mortality rate” using R software version 3.6.3.

The Akaike information criterion (AIC) was used to select the best model with which to compute the predicted national under-five mortality rate [38]. This applied to the unweighted model, since the STATA software that was used to fit the models does not produce AIC values for weighted models. The initial model included all variables listed in Section “Data”. The second model dropped the variables whose coefficients had large *p*-values in the first model.

## Results

The data summary in Table 1 shows that cases of deaths of children aged below five years have been lower than 10% in Malawi during the 15 years prior to 2016. In addition, the proportions of under-five deaths showed a decreasing trend during this period, such that the percentage was almost halved between 2010 and 2016. Further, the data showed that the percentages of under-five deaths were higher in male babies, birth order of 1 or 6 and above, caesarean births, twin or multiple births, and in home-based deliveries, across all the three surveys. Similarly, the majority of under-five deaths were observed in babies who were born to mothers aged either below 20 years or 35 years and above, to those whose preceding birth interval was less than 24 months, others who were not using modern contraceptive methods, those who had no formal educational qualification, and others who were working. Furthermore, the percentage of under-five deaths was higher in children from rural areas, and children from central and southern regions of the study country. The Chi-square test of independence showed that all the studied explanatory variables had individual significant association with the under-five child death variable, evidenced by at least two of the MDHS data sets.

**Table 1:** Distribution of the sample and observed under-five death cases over socio-demographic variables and the association of each variable with the death outcome

	2004 MDHS			2010 MDHS			2015 MDHS		
Variable	n (%)	Died (%)	$\boldsymbol\chi^{\mathbf2}$ *p*-val	n (%)	Died (%)	$\boldsymbol\chi^{\mathbf2}$ *p*-val	n (%)	Died (%)	$\boldsymbol\chi^{\mathbf2}$ *p*-val
Overall sample	10,914 (100)	1,056 (9.7)		19,967 (100)	1,607 (8.1)		17,286 (100)	824 (4.8)	
Child’s Sex			$<0.0001$			0.001			0.003
Male	5,523 (50.6)	593 (10.7)		9,979 (50.0)	865 (8.7)		8,687 (50.3)	455 (5.2)	
Female	5,391 (49.4)	463 (8.6)		9,988 (50.0)	742 (7.4)		8,599 (49.8)	369 (4.3)	
Birth order			$<0.0001$			$<0.0001$			$<0.0001$
1	2,469 (22.6)	290 (11.8)		3,925 (19.7)	363 (9.3)		4,400 (25.5)	267 (6.1)	
2-5	6,403 (58.7)	566 (8.8)		12,049 (60.3)	876 (7.3)		10,370 (60.0)	414 (4.0)	
6+	2,042 (18.7)	200 (9.8)		3,993 (20.0)	368 (9.2)		2,516 (14.6)	143 (5.7)	
Caesarean birth			0.641			0.012			0.006
No	10,575 (97.0)	1,021 (9.7)		19,002 (95.4)	1,507 (7.9)		16,122 (93.5)	749 (4.7)	
Yes	326 (3.0)	34 (10.4)		907 (4.6)	93 (10.3)		1,116 (6.5)	72 (6.5)	
Delivery type			$<0.0001$			$<0.0001$			$<0.0001$
Singleton	10,547 (96.6)	938 (8.9)		19,104 (95.7)	1,396 (7.3)		16,618 (96.1)	707 (4.3)	
Multiple	367 (3.4)	118 (32.2)		863 (4.3)	211 (24.5)		668 (3.9)	117 (17.5)	
Birth place			0.013			0.482			0.007
Home or other	3,205 (29.7)	343 (10.7)		4,934 (25.5)	400 (8.1)		1,354 (7.8)	85 (6.3)	
Health facility	7,576 (70.3)	694 (9.2)		14,446 (74.5)	1,126 (7.8)		15,932 (92.2)	739 (4.6)	
Maternal age			0.161			$<0.0001$			$<0.0001$
<20	713 (6.5)	80 (11.2)		1,179 (5.9)	102 (8.7)		1,246 (7.2)	85 (6.8)	
20-34	8,254 (75.6)	775 (9.4)		14,792 (74.1)	1,109 (7.5)		12,647 (73.2)	558 (4.4)	
35-49	1,947 (17.8)	201 (10.3)		3,996 (20.0)	396 (9.9)		3,393 (19.6)	181 (5.3)	
Preceding birth-interval			$<0.0001$			$<0.0001$			$<0.0001$
<24 months	1,538 (18.3)	218 (14.2)		2,281 (14.3)	306 (13.4)		1,446 (11.3)	113 (7.8)	
24 to 36 months	2,994 (35.6)	252 (8.4)		6,327 (39.5)	429 (6.8)		3,705 (28.9)	169 (4.6)	
> 36 months	3,890 (46.2)	291 (7.5)		7,400 (46.2)	503 (6.8)		7,690 (59.9)	264 (3.4)	
Contraceptive use			$<0.0001$			$<0.0001$			$<0.0001$
Not using	7,957 (72.9)	863 (10.9)		11,484 (57.5)	1,037 (9.0)		7,021 (40.6)	436 (6.2)	
Using	2,957 (27.1)	193 (6.5)		8,483 (42.5)	570 (6.7)		10,265 (59.4)	388 (3.8)	
Maternal educ			$<0.0001$			0.002			0.076
No education	2,870 (26.3)	308 (10.7)		3,372 (16.9)	289 (8.6)		2,161 (12.5)	101 (4.7)	
Primary	6,967 (63.8)	678 (9.7)		13,865 (69.4)	1,145 (8.3)		11,456 (66.3)	573 (5.0)	
Secondary or above	1,077 (9.9)	70 (6.5)		2,730 (13.7)	173 (6.3)		3,669 (21.2)	150 (4.1)	
Maternal job status			0.767			0.026			0.013
Not working	4,449 (40.8)	435 (9.8)		8,194 (41.1)	617 (7.5)		5,938 (34.4)	250 (4.2)	
Working	6,464 (59.2)	621 (9.6)		11,733 (58.9)	986 (8.4)		11,348 (65.7)	574 (5.1)	
Residence			$<0.0001$			0.696			0.033
Urban	1,137 (10.4)	76 (6.7)		1,896 (9.5)	157 (8.3)		2,766 (16.0)	110 (4.0)	
Rural	9,777 (89.6)	980 (10.0)		18,071 (90.5)	1,450 (8.0)		14,520 (84.0)	714 (4.9)	
Region			0.049			$<0.0001$			0.018
Northern	1,349 (12.5)	106 (7.9)		3,560 (17.8)	232 (6.5)		3,208 (18.6)	126 (3.9)	
Central	4,141 (37.9)	418 (10.1)		6,866 (34.4)	544 (7.9)		6,023 (34.8)	316 (5.3)	
Southern	5,424 (49.7)	532 (9.8)		9,541 (47.8)	831 (8.7)		8,055 (46.6)	382 (4.7)	

The results in Table 2 provide the model estimates upon including all the available covariates from Table 1 to describe under-five child death. It is shown, in both unweighted and weighted models and across all the surveys, that the adjusted odds of death of the under-five child were lower in female children, preceding birth interval of 24 months and above, and in children born to mothers that used modern contraceptive methods. The adjusted odds of death were higher in caesarean births, twin or multiple delivery births, children born to working class mothers, those born to mothers aged 35 to 49 years, children from rural areas, and those born in central and southern regions. The effects of birth order, place of delivery, and place of residence on child death outcome were not statistically significant in a model that had the other variables mentioned above. Hence, these covariates were dropped in the final model that was used to predict the under-five mortality rate and estimate the bias. The AIC values in Table 2 were reserved for comparisons with those obtained upon excluding the mentioned variables.

**Table 2:** Effects of child characteristics on death outcome upon fitting full logit model to MDHS data

	Unweighted Full Logit Model			Weighted Full Logit Model		
	2004 DHS	2010 DHS	2015 DHS	2004 DHS	2010 DHS	2015 DHS
Variable	aOR (*p*-value)	aOR (*p*-value)	aOR (*p*-value)	aOR (*p*-value)	aOR (*p*-value)	aOR (*p*-value)
Child’s Sex						
Male$*$						
Female	0.75 ($<0.0001$)	0.85 (0.007)	0.81 (0.019)	0.82 (0.025)	0.86 (0.030)	0.80 (0.016)
Birth order						
1$*$						
2-5	1.24 (0.076)	1.14 (0.189)	0.93 (0.582)	1.25 (0.067)	1.09 (0.325)	0.91 (0.492)
6+	0.80 (0.067)	0.92 (0.325)	1.09 (0.492)	0.81 (0.076)	0.88 (0.189)	1.08 (0.582)
Caesarean birth						
No$*$						
Yes	1.32 (0.249)	1.46 (0.010)	1.65 (0.003)	1.18 (0.633)	1.35 (0.077)	1.64 (0.011)
Delivery type						
Singleton $*$						
Multiple	5.62 ($<0.0001$)	4.62 ($<0.0001$)	4.78 ($<0.0001$)	5.90 ($<0.0001$)	4.48 ($<0.0001$)	4.69 ($<0.0001$)
Birth place						
Home or other $*$						
Health facility	0.99 (0.951)	1.05 (0.482)	0.81 (0.129)	1.07 (0.507)	1.02 (0.809)	0.82 (0.205)
Maternal age						
<20 $*$						
20-34	1.04 (0.903)	1.50 (0.277)	1.18 (0.751)	0.99 (0.975)	1.35 (0.418)	1.21 (0.729)
35-49	1.42 (0.345)	2.24 (0.033)	1.56 (0.406)	1.39 (0.406)	1.97 (0.070)	1.54 (0.453)
Preceding birth-interval						
<24 months $*$						
24 to 36 months	0.54 ($<0.0001$)	0.46 ($<0.0001$)	0.55 ($<0.0001$)	0.55 ($<0.0001$)	0.47 ($<0.0001$)	0.54 ($<0.0001$)
> 36 months	0.44 ($<0.0001$)	0.43 ($<0.0001$)	0.40 ($<0.0001$)	0.43 ($<0.0001$)	0.47 ($<0.0001$)	0.39 ($<0.0001$)
Contraceptive use						
Not using $*$						
Using	0.65 ($<0.0001$)	0.71 ($<0.0001$)	0.61 ($<0.0001$)	0.63 (0.0001)	0.71 ($<0.0001$)	0.58 ($<0.0001$)
Maternal educ						
No education $*$						
Primary	0.93 (0.435)	1.06 (0.452)	1.16 (0.258)	0.91 (0.323)	1.02 (0.835)	1.18 (0.249)
Secondary or above	0.60 (0.815)	0.91 (0.447)	1.08 (0.663)	0.71 (0.189)	0.80 (0.145)	1.10 (0.621)
Maternal job status						
Not working $*$						
Working	1.02 (0.815)	1.10 (0.139)	1.4117 (0.001)	1.02 (0.857)	1.10 (0.203)	1.39 (0.004)
Residence						
Urban $*$						
Rural	1.24 (0.171)	0.87 (0.226)	1.28 (0.101)	1.32 (0.165)	0.91 (0.448)	1.26 (0.173)
Region						
Northern $*$						
Central	1.12 (0.419)	1.23 (0.039)	1.1974 (0.177)	1.15 (0.374)	1.33 (0.008)	1.17 (0.265)
Southern	1.16 (0.290)	1.44 ($<0.0001$)	1.08 (0.535)	1.29 (0.101)	1.55 ($<0.0001$)	1.11 (0.430)
AIC	4786.21	7904.79	4285.79	-	-	-

aOR = adjusted odds ratio; $``*''$ = reference level; $``-''$ implied weighted model output did not include AIC nor log-likelihood value

Upon fitting the reduced logit models to the datasets, it is shown in Table 3 that the AIC values did not change much compared to those found before. Trying to drop each of the three covariates independently worsened the fit of the models, hence the results of the ML estimates in Table 3 for models without the three stated covariates were used for computation of the bias in this study. The results in Table 3 showed that the *p*-values for the reduced models were lower compared to the ones given in Table 2. But the sizes and directions of the estimates did not change. The odds of death of the under-five child were significantly lower in female compared to male children, those born to mothers who had preceding birth interval of 24 months and above, children born to mothers who used modern contraceptive methods, and those whose mother’s highest level of education was secondary and above. Whereas the odds of child death were higher in caesarean births, twin or multiple births, children born to mothers aged 35 to 49 years, those born to mothers who were working, and those from central and southern regions. In addition, both the ML estimates and *p*-values adjusted slightly upwards or downwards in the weighted compared to unweighted models, which showed some bias in the estimates of the unweighted models.

**Table 3:** Effects of child characteristics on death outcome upon fitting reduced logit model to MDHS data

	Unweighted Reduced Logit Model			Weighted Reduced Logit Model		
	2004 DHS	2010 DHS	2015 DHS	2004 DHS	2010 DHS	2015 DHS
Variable	aOR (*p*-value)	aOR (*p*-value)	aOR (*p*-value)	aOR (*p*-value)	aOR (*p*-value)	aOR (*p*-value)
Child’s Sex						
Male$*$						
Female	0.74 ($<0.0001$)	0.84 (0.004)	0.81 (0.021)	0.80 (0.014)	0.86 (0.022)	0.80 (0.017)
Birth order						
1$*$						
2-5	-	-	-	-	-	-
6+	-	-	-	-	-	-
Caesarean birth						
No$*$						
Yes	1.30 (0.282)	1.47 (0.009)	1.56 (0.008)	1.18 (0.631)	1.35 (0.076)	1.56 (0.022)
Delivery type						
Singleton $*$						
Multiple	5.51 ($<0.0001$)	4.58 ($<0.0001$)	4.83 ($<0.0001$)	5.82 ($<0.0001$)	4.43 ($<0.0001$)	4.73 ($<0.0001$)
Birth place						
Home or other $*$						
Health facility	-	-	-	-	-	-
Maternal age						
<20 $*$						
20-34	1.03 (0.932)	1.52 (0.258)	1.20 (0.725)	0.98 (0.953)	1.38 (0.390)	1.23 (0.704)
35-49	1.21 (0.593)	2.22 (0.033)	1.66 (0.334)	1.18 (0.667)	1.93 (0.076)	1.63 (0.380)
Preceding birth-interval						
<24 months $*$						
24 to 36 months	0.54 ($<0.0001$)	0.46 ($<0.0001$)	0.55 ($<0.0001$)	0.57 ($<0.0001$)	0.47 ($<0.0001$)	0.54 ($<0.0001$)
> 36 months	0.45 ($<0.0001$)	0.42 ($<0.0001$)	0.39 ($<0.0001$)	0.45 ($<0.0001$)	0.46 ($<0.0001$)	0.38 ($<0.0001$)
Contraceptive use						
Not using $*$						
Using	0.64 ($<0.0001$)	0.72 ($<0.0001$)	0.60 ($<0.0001$)	0.63 ($<0.0001$)	0.72 ($<0.0001$)	0.58 ($<0.0001$)
Maternal educ						
No education $*$						
Primary	0.94 (0.441)	1.11 (0.177)	1.12 (0.369)	0.92 (0.391)	1.08 (0.419)	1.14 (0.337)
Secondary or above	0.58 (0.007)	0.95 (0.671)	1.43 ($<0.0001$)	0.69 (0.139)	0.85 (0.250)	0.98 (0.922)
Maternal job status						
Not working $*$						
Working	1.02 (0.773)	1.08 (0.199)	1.43 ($<0.0001$)	1.03 (0.754)	1.08 (0.279)	1.41 (0.003)
Residence						
Urban $*$						
Rural	-	-	-	-	-	-
Region						
Northern $*$						
Central	1.12 (0.420)	1.25 (0.023)	1.19 (0.190)	1.14 (0.419)	1.37 (0.003)	1.16 (0.271)
Southern	1.13 (0.356)	1.47 ($<0.0001$)	1.08 (0.552)	1.24 (0.172)	1.59 ($<0.0001$)	1.11 (0.438)
AIC	4870.06	8268.38	4285.64	-	-	-

aOR = adjusted odds ratio; $``*''$ = reference level; $``-''$ in AIC row implied weighted model output did not include AIC nor log-likelihood value; “Reduced” Logit Model implied a model with some covariates that formed part of the initial model dropped in this second model

The results for bias of under-five mortality rate estimate are given in Table 4. It is shown that the bias was smaller in the weighted compared to the unweighted predicted under-five mortality rate, regardless of whether the fitted model was weighted or not. Further, the results showed that the standard errors were also smaller for the weighted than the unweighted predicted mortality rate, irrespective of the weighting status of the model. In addition, it is shown that the bias estimate for the under-five mortality rate decreased in each year of survey, such that it was smallest in 2015-16 MDHS. Furthermore, the results showed that the bias estimates of weighted predicted mortality from the 2010 MDHS data increased slightly with a decreasing sample size, but this was not always the case with the 2004 and 2015 surveys, as there were smaller bias estimates even with smallest sample sizes for the MDHS data in the stated years. With the unweighted predicted mortality rate, the bias estimates were insensitive of sample size, for all the three surveys. Finally, the standard errors of the weighted predicted mortality rate remained the same upon reducing sample size, but they increased slightly in the case of unweighted predicted mortality.

**Table 4:** Bias in under-five mortality estimate using unweighted and weighted logit model, as well as unweighted and weighted predicted mortality estimate

n	$\widehat{\boldsymbol\pi}\boldsymbol(\mathbf x\boldsymbol)\boldsymbol(\boldsymbol S\boldsymbol.\boldsymbol E\boldsymbol.\boldsymbol)$	$\boldsymbol\pi$	$\boldsymbol b\boldsymbol i\boldsymbol a\boldsymbol s\boldsymbol(\widehat{\boldsymbol\pi}\boldsymbol(\textbf{x}\boldsymbol)\boldsymbol)$	$\widehat{\boldsymbol\pi}\boldsymbol(\mathbf x\boldsymbol)\boldsymbol(\boldsymbol S\boldsymbol.\boldsymbol E\boldsymbol.\boldsymbol)$	$\boldsymbol\pi$	$\boldsymbol b\boldsymbol i\boldsymbol a\boldsymbol s\boldsymbol(\widehat{\boldsymbol\pi}\boldsymbol(\textbf{x}\boldsymbol)\boldsymbol)$
	2004 unweighted model, unweighted $\hat{\pi }(\textbf{x})$			2004 weighted model, weighted $\hat{\pi } (\textbf{x})$		
10,914	0.004 (0.001)	0.133	**-0.129**	0.086 (0.000)	0.133	**-0.047**
8,292	0.004 (0.001)	0.133	**-0.129**	0.091 (0.000)	0.133	**-0.042**
5,624	0.004 (0.001)	0.133	**-0.129**	0.087 (0.000)	0.133	**-0.046**
2,878	0.004 (0.001)	0.133	**-0.129**	0.089 (0.000)	0.133	**-0.044**
	2004 unweighted model, weighted $\hat{\pi }(\textbf{x})$			2004 weighted model, unweighted $\hat{\pi } (\textbf{x})$		
10,914	0.090 (0.000)	0.133	**-0.043**	0.004 (0.001)	0.133	**-0.129**
8,292	0.091 (0.000)	0.133	**-0.042**	0.004 (0.001)	0.133	**-0.129**
5,624	0.089 (0.000)	0.133	**-0.044**	0.004 (0.001)	0.133	**-0.129**
2,878	0.090 (0.000)	0.133	**-0.043**	0.004 (0.001)	0.133	**-0.129**
	2010 unweighted model, unweighted $\hat{\pi }(\textbf{x})$			2010 weighted model, weighted $\hat{\pi }(\textbf{x})$		
19,967	0.003 (0.000)	0.112	**-0.109**	0.077 (0.000)	0.112	**-0.035**
15,064	0.003 (0.001)	0.112	**-0.109**	0.076 (0.000)	0.112	**-0.036**
10,153	0.003 (0.001)	0.112	**-0.109**	0.074 (0.000)	0.112	**-0.038**
5,030	0.003 (0.001)	0.112	**-0.109**	0.070 (0.000)	0.112	**-0.042**
	2010 unweighted model, weighted $\hat{\pi }(\textbf{x})$			2010 weighted model, unweighted $\hat{\pi }(\textbf{x})$		
19,967	0.077 (0.000)	0.112	**-0.035**	0.003 (0.000)	0.112	**-0.109**
15,064	0.077 (0.000)	0.112	**-0.035**	0.003 (0.001)	0.112	**-0.109**
10,153	0.075 (0.000)	0.112	**-0.037**	0.003 (0.001)	0.112	**-0.109**
5,030	0.072 (0.000)	0.112	**-0.040**	0.003 (0.001)	0.112	**-0.109**
	2015 unweighted model, unweighted $\hat{\pi }(\textbf{x})$			2015 weighted model, weighted $\hat{\pi }(\textbf{x})$		
17,286	0.002 (0.000)	0.063	**-0.061**	0.040 (0.000)	0.063	**-0.023**
13,099	0.002 (0.000)	0.063	**-0.061**	0.038 (0.000)	0.063	**-0.025**
8,644	0.002 (0.001)	0.063	**-0.061**	0.038 (0.000)	0.063	**-0.025**
4,314	0.002 (0.001)	0.063	**-0.061**	0.045 (0.000)	0.063	**-0.018**
	2015 unweighted model, weighted $\hat{\pi }(\textbf{x})$			2015 weighted model, unweighted $\hat{\pi }(\textbf{x})$		
17,286	0.042 (0.000)	0.063	**-0.021**	0.002 (0.000)	0.063	**-0.061**
13,099	0.039 (0.000)	0.063	**-0.024**	0.002 (0.000)	0.063	**-0.061**
8,644	0.040 (0.000)	0.063	**-0.023**	0.002 (0.001)	0.063	**-0.061**
4,314	0.046 (0.000)	0.063	**-0.017**	0.002 (0.001)	0.063	**-0.061**

$\hat{\pi }(\textbf{x})$ = overall predicted under-five mortality rate; *S*.*E*. = standard error of $\hat{\pi }(\textbf{x})$;

$\pi$= raw under-five mortality rate from MDHS report; $bias(\hat{\pi }(\textbf{x}))$=$\hat{\pi }(\textbf{x})-\pi$

## Discussion

In this paper, the usefulness of survey weights in the prediction of under-five mortality through regression methods was investigated. The results showed that weighting of the model causes some positive and negative changes in the maximum likelihood (ML) estimates that were originally obtained from the unweighted model. This confirms the presence of some bias in ML estimates for unweighted models [11, 36]. However, the study has found that ignoring the sampling weights during model fitting has little impact on the accuracy of estimation of the overall predicted under-five mortality rate. It is the survey design weighting that is applied on the predicted mortality estimates from each cluster that has a significant effect on the bias of the overall predicted under-five mortality rate. This implies that the up and down shifts in the ML estimates for various explanatory variables cancel the weighting effect during the computations of the fitted probabilities of events for individuals, which are used when calculating the overall predicted mortality rate. Hence, the need for additional cluster-strata weights on the cluster predicted mortality rates in order to achieve a low-biased overall predicted under-five mortality estimate.

The findings showed that the biases in mortality estimate decreased with each survey year, where the bias was smallest when using the 2015-16 MDHS data to predict the prevailing under-five mortality compared to previous surveys. This reflected improvements in data quality assurance measures that the Measure DHS programme and National Statistics Office have been making over the years. For example, the 2015-16 MDHS data were collected using the computer-assisted personal interviews (CAPI) tool, unlike in the 2004 MDHS where questionnaires were administered physically and data entered manually. This manual process of managing data could be the reason why some variables like birth weight, had more missing values in the 2004 MDHS compared to the 2015-16 MDHS data. Missing data have potential for disturbing the randomness and representativeness of the sample, while increasing the bias in the overall mortality estimates. In addition, the household geo-coordinates data were pre-recorded ahead of the survey and used to guide the enumerators so they could reach the right sampled households and avoid unauthorised swapping of interviewees during the 2015-16 MDHS. Furthermore, the households in semi-urban locations were re-classified into right rural or urban strata during the 2015-16 MDHS, so that a child’s data could be gathered correctly. This was unlike the previous surveys where such households could wrongly be assigned to urban strata. Therefore, these initiatives led to a high response rate and good data quality in the 2015-16 MDHS compared to 2010 and 2004 MDHSs. In turn, this led to reduced bias of predicted under-five mortality rate for the 2015-16 MDHS, as observed in the present study [30].

The study has also shown that reducing the sample size of the MDHS only led to a slight increase in standard errors of the unweighted predicted mortality rates, and not in the sizes of the estimates themselves, as they stayed constant despite the lowering of the sample size. It has been observed that a sample size of as low as 2% of the population of all children in the country could still yield low-biased mortality estimates, provided that the sample was randomly selected and representative of the population, and that the overall predicted mortality rate was weighted. This was expected as the unweighted variance measure for sample proportion such as unweighted mortality rate involved a total sample size in the denominator, which inflated the variance in lower sample sizes. But this could not affect the value of mortality estimate due to the effects of sample randomness and representativeness. When a researcher has no access to primary survey data but aggregated data from two or more surveys for a country or region to which they wish to make similar predictions, then methods of pooled and weighted survey estimation using mixed-effects hierarchical models or meta-analysis could give reliable estimates provided all the pooled surveys were conducted within the same time of inference [4, 10].

The model’s ML estimates showed that the risk of under-five mortality was lower in female compared to male children, in children whose mothers had preceding birth interval of 24 months and above, children whose mothers used modern contraceptive methods, and those whose mothers attended up to secondary education and above. The risk was higher in caesarean births, twin or multiple births, in children whose mothers were aged 35 to 49 years, children born to working class mothers, and children residing in central and southern regions of Malawi. These results are consistent with previous findings, and there are also clear biological and social explanations in literature for these observations [18, 26, 37, 45, 47]. For example, the well-educated mothers have an upper hand in terms of knowledge and skills about healthcare, which benefits the baby’s health compared to the less-educated mothers [26]. The high risk of death in twin births largely reflects an increased risk of intrapartum anoxia in the second twin born at term that reduce their chances of survival [47]. With the caesarean births, the high risk of death stems from increased chances of iatrogenic prematurity or respiratory disease [45]. While the lower death rates in female babies is attributed to their genetic and biological make-up together with preconception environments that lowers their risk against most diseases compared to male babies [37]. Furthermore, the high risk of under-five deaths in children from working class mothers is attributed to inadequate breastfeeding that their babies are subject to [2].

## Conclusion

This paper investigated the impact of survey sampling design on the predicted under-five mortality rate using regression methods applied on three recent demographic and health survey data in Malawi. The findings showed that the risk factors for under-five mortality have not changed from those observed in previous studies in the sub-Saharan African region and other Low and Middle Income Countries (LMIC). The study has found that the model’s probability weights have very little effect on the bias of the predicted mortality rate, so that ignoring the weighting during the model fitting does not change the predicted mortality value. However, it has been observed that weighting the cluster-specific predicted mortality rates has significant effects in minimising the bias of the overall predicted under-five mortality rate. The study has also found that the bias estimates for prevailaing under-five mortality rates decreased with each survey year, resulting in much lower-biased estimates seen in the 2015-16 MDHS than in previous surveys. Furthermore, it has been observed that a random and representative sample size of at least 2% of the population is enough to obtain low-biased under-five mortality estimates, provided the computation of overall mortality estimate considers cluster weights. A small sample size only affects the standard errors of an estimated mortality, which become large and widen confidence intervals for the estimates.

This study therefore recommends applying cluster sampling weights in cluster-specific predicted probabilities of event for calculating overall predicted probability of the event, when analysing binary health outcomes through regression methods, without regard of weighting the model fitting process. Although this study focused on models for binary response variable, the findings apply to other models for categorical variables with more than two levels. Since the reliability of regression methods rely on appropriateness of the data for the included covariates in the models, further improvements in DHS data quality control techniques will help in yielding more accurate predictions of under-five mortality rate through regression-based methods proposed in this study. Future research could incorporate methods for addressing missing data in explanatory variables such as birth weight and antenatal care visits, so that the models use as many covariates as possible while observing the effect of sampling design in predicting under-five mortality rate.

## Data Availability

The MDHS data that were used in this study are publicly and freely available for users at https://dhsprogram.com/data/new-user-registration.cfm.
